# Hypermucoviscous Multidrug-Resistant *Klebsiella variicola* Strain LL2208 Isolated from Chinese Longsnout Catfish (*Leiocassis longirostris*): Highly Similar to Human *K. variicola* Strains

**DOI:** 10.3390/pathogens13080647

**Published:** 2024-07-31

**Authors:** Qingyong Li, Xin Yu, Lin Ye, Tongyu Hou, Yi Liu, Guiming Liu, Qing Wang, Defeng Zhang

**Affiliations:** 1 Fisheries Research and Extension Center of Huizhou, Huizhou 516055, China; byqyli@163.com (Q.L.); 27763967@126.com (X.Y.); 13528069240@139.com (L.Y.); houtongyu@hotmail.com (T.H.); 3825431221@139.com (Y.L.); 13928333187@189.com (G.L.); 2 Key Laboratory of Fishery Drug Development, Ministry of Agriculture and Rural Affairs, Pearl River Fisheries Research Institute, Chinese Academy of Fishery Sciences, Guangzhou 510380, China; wangqing@prfri.ac.cn; 3 Guangdong Provincial Key Laboratory of Aquatic Animal Immunology and Sustainable Aquaculture, Pearl River Fisheries Research Institute, Chinese Academy of Fishery Sciences, Guangzhou 510380, China

**Keywords:** *Klebsiella variicola*, multidrug resistant strain, genome, zoonotic transmission, freshwater fish

## Abstract

Outbreaks of bacterial diseases occur in farmed Chinese longsnout catfish (*Leiocassis longirostris*). Due to limited information on aquatic *Klebsiella* variicola-infected animals, this study aimed to identify strain LL2208 isolated from diseased *L. longirostris*, determine its biological features, and evaluate its risk to public health. Strain LL2208 was tested for molecular identification, challenge, string, biofilm formation, and antimicrobial susceptibility. Furthermore, the whole genome of the strain was sequenced and analyzed. Based on molecular identification, strain LL2208 was identified as *K. variicola*. Artificial infection showed that this strain was moderately virulent to *L. longirostris* with an LD_50_ = 7.92 × 10^7^ CFU/mL. Antibiotic sensitivity tests showed that this strain was resistant to penicillins, macrolides, aminoglycosides, amphenicols, glycopeptides, and lincosamide, indicating multidrug resistance. Strain LL2208 has a genome size of 5,557,050 bp, with a GC content of 57.38%, harboring 30 antimicrobial resistance genes and numerous virulence-related genes. Its molecular type was ST595-KL16-O5. Collinearity analysis showed that strain LL2208 was highly similar to the human-derived *K. variicola* strain. In conclusion, the multidrug-resistant and virulent *K. variicola* strain LL2208 was isolated from fish and may have originated from humans. These results provide a foundation for further studies on the transmission of *K. variicola* between humans and aquatic animals.

## 1. Introduction

The *Klebsiella* genus belongs to the Enterobacteriaceae family, and its most common pathogenic member is *Klebsiella pneumoniae*. *K. pneumoniae* strains are divided into three phylogenetic groups: KpI (*K. pneumoniae*), KpII (*Klebsiella quasipneumoniae*), and KpIII (*Klebsiella variicola*) [1,2]. *K. pneumoniae* is the most prevalent cause of respiratory tract infection in humans (21.1%) [3], and the resistance rates to third-generation cephalosporins, such as cefotaxime and ceftriaxone, are 24.4% and 22.7%, respectively. Additionally, *K. pneumoniae* is a pathogenic bacterium in aquatic animals, for example, large yellow croaker (*Larimichthys crocea*) [4], *Megalobrama amhlycephala* [5], largemouth bass (*Micropterus salmoides*) [6], *Procambarus clarkia* [7], *Anguilla marmorata* [8], giant salamander (*Andrias davidianus*) [9], Nile tilapia (*Oreochromis niloticus*) [10], and American bullfrog (*Rana catesbeiana*) [11].

*K. variicola* is an opportunistic pathogen that can cause severe infections in humans and animals [12]. It is an emerging human pathogen that causes hospital- and community-acquired infections, including respiratory tract, bloodstream, urinary tract, and endodontic infections [13,14,15]. A multidrug-resistant and hypermucoviscous *K. variicola* (hmvKv) strain was isolated from a pediatric patient [16]. *K. variicola* has been reported to infect farmed animals, including horses with respiratory diseases [17] and dead chicken embryos [1]. However, limited information is available regarding aquatic animals infected with *K. variicola* [18].

Antibiotics play an important role in preventing and treating bacterial infections. Recently, the reports on extended-spectrum-*β*-lactamase- or carbapenemase-producing *K. variicola* strains have been increasing [19], such as the *K. variicola* strain 4717 harboring *bla*_OXA-484_, which was found in China [20], and *K. variicola* strain SHET-01 containing the coexisting of *bla*_NDM-1_ and *bla*_IMP-4_ genes [16], while *K. variicola* strain 18L carries *bla*_LEN-21_ and *bla*_LEN-24_ genes in the chromosome [15]. A polymyxin-resistant *K. variicola* strain, M50, with a non-functional *mgrB* variant, was isolated from a bird in Brazil [21]. The *LEN-17*, *oqxA*, *oqxB*, *fosA*, and *fosA7* genes have been identified in the genome of *K. variicola* strain F2R9 (=ATCC BAA-830) isolated from banana root [22]. *K. variicola* strain KV093 was isolated from a urine sample in India, and the *LEN-9* and *fosA6* genes were contained in its genome [23]. Plasmids play an important role in the horizontal transfer of antimicrobial-resistant genes in *K. variicola* strains [12]. Therefore, *K. variicola* strains containing numerous antimicrobial resistance genes pose a public health challenge. Consequently, it is important to determine the distribution and genetic backgrounds of multidrug-resistant *K. variicola* strains.

The Chinese longsnout catfish (*Leiocassis longirostris*) belongs to the Bagridae family and is a commercially important freshwater fish in China distributed in the Yangtze, Minjiang, Pearl, Liaohe, and Huaihe Rivers [24]. Intensive farming of this fish has been widely promoted because of its good taste, lack of intermuscular bone, high market demand, and high price [25]. The annual production of *L. longirostris* exceeded 20,000 tons in 2020 [26], and the production is continuously increasing. It has a naturally high disease resistance [26]. However, with the increased aquaculture density in ponds and the continuous water quality deterioration, many bacterial diseases frequently occur during farming, including ulcerative syndrome caused by *Aeromonas veronii* [27] and edwardsiellosis caused by *Edwardsiella ictaluri* [28] and bacterial gill-rot disease caused by *Flavobacterium columnare*.

Recently, high-density farmed *L. longirostris* disease outbreaks were reported in Foshan City, Guangdong province, China. A pathogen named strain LL2208 was isolated from the diseased fish. This study aimed to identify strain LL2208 isolated from diseased *L. longirostris* and evaluate its risk to public health by analyzing its biological features and genetic characteristics. Strain LL2208 was identified as *K. variicola*, and it was similar to *K. variicola* isolated from humans. To the best of our knowledge, this is the first report on multidrug-resistant *K. variicola* from fish.

## 2. Materials and Methods

### 2.1. Bacterial Isolation and Identification

Naturally diseased Chinese longsnout catfish samples (N = 7) were collected from a farm (three ponds) in Foshan City, Guangdong Province, China. The water temperature was 29 to 32 °C, and the cumulative mortality rate was 15–40% within 20 d. The liver, spleen, and kidney tissues were collected and streaked on a Columbia blood agar plate (Guangzhou Detgerm Microbiogical Science Ltd., Guangzhou, China) and then incubated at 29 °C for 48 h. The dominant colonies were purified by streaking and re-streaking on brain heart infusion (BHI; Becton, Dickinson and Company, Sparks, MD, USA) agar plates. One of the representative strains (LL2208) was selected and inoculated on a Columbia blood agar plate at 28 °C for 24 h to observe the colony morphology. An electron microscopy negative-staining sample of strain LL2208 was prepared to observe the bacterial characteristics.

Strain LL2208 was inoculated into a BHI liquid medium for expansion. The bacterial cells were collected, and the genomic DNA was extracted using a bacterial genomic DNA extraction kit (Guangzhou Magen Biotechnology, Co., Ltd., Guangzhou, China). The 16S rRNA gene of strain LL2208 was PCR amplified using the universal primers 27f (5′-AGAGTTTGATCMTGGCTCAG-3′) and 1492r (5′-TACGGYTACCTTGTTACGACT-3′), and the positive PCR product was sequenced. The 16S rRNA gene was analyzed using Blastn. Further, a phylogenetic tree was constructed using MEGA v7.0.20 with Neighbor Joining (Bootstrap 1000 replicates), and species with distant genetic relationships, such as *Citrobacter freundii* and *Plesiomonas shigelloides*, were selected as outgroups. The one-step multiplex PCR was performed to determine the taxonomic status [29]. The primers LEN-F and DeoR-R were used, and PCR was performed using the Ex Taq™ Version 2.0 plus dye (Takara Bio, Shiga, Japan) according to the manufacturer’s instructions. The PCR amplification conditions were as follows: pre-denaturation at 95 °C for 4 min, followed by 35 cycles (denaturation at 94 °C for 30 s, annealing at 56 °C for 30 s, and extension at 72 °C for 1 min), and final extension at 72 °C for 10 min. Amplified products were collected and sequenced by Sangon Biotech (Shanghai) Co., Ltd., Shanghai, China.

### 2.2. String Test

Strain LL2208 was streaked onto a MacConkey agar (Guangdong HuanKai Microbial Sci. & Tech. Co., Ltd., Guangzhou, China) plate and incubated at 30 °C for 18 h. The length of the viscous filament was observed by making contact with the colony surface using the inoculum ring, with a positive strain being ≥10 mm and a negative strain being <10 mm [12]. Strain LL2208 was passed twice, and the string test was repeated. The strain that tested positive on the string test could therefore be classified as hmvKv.

### 2.3. Biofilm Formation Assay

The biofilm formation ability of strain LL2208 was evaluated as previously described, with slight modifications [30]. The strain was inoculated briefly into Luria–Bertani (LB) medium (Becton, Dickinson and Company, Sparks, MD, USA), cultured until it reached the logarithmic growth phase (OD_600_ = 0.8), and then the bacterial culture was diluted 1:100 in 5 mL of fresh LB broth. Subsequently, 200 μL of the diluted bacterial cultures and 200 μL of LB broth were added, respectively, to each well of 96-well polystyrene microtiter plates and each well in the control group. The 96-well plates were incubated at 35 °C for 24 h. The supernatant was discarded, the wells were washed thrice with sterile water, and methanol solution (200 μL/well) was added to fix the biofilm for 15 min. The methanol solution was discarded, and the 96-well plates were dried for 15 min. The crystal violet solution was added to each well (200 μL/well) to stain for 10 min at room temperature (25 °C), and the excess stain was removed three times with sterile water. Acetic acid (33%, *v*/*v*) was added to each well to re-solubilize the attached stain after drying in a 96-well plate. The OD_595_ was measured using a microplate reader. The biofilm assay was performed in triplicate.

Biofilm formation was categorized into weak, moderate, or strong according to the absorbance value: weak biofilm producer, OD_595c (control group)_ < OD_595_ ≤ 2 OD_595c_; moderate biofilm producer, 2 OD_595c_ < OD_595_ ≤ 4 OD_595c_; strong biofilm producer, OD_595_ ≥ 4 OD_595c_.

### 2.4. Challenge Test

Strain LL2208 was inoculated into BHI medium and cultured at 28 °C until the logarithmic growth phase (OD_600_ = 0.8–1.0). The bacterial cells were collected by centrifugation at 5000 rpm, and the bacterial concentration was adjusted to 5.0 × 10^8^, 5.0 × 10^7^, 5.0 × 10^6^, 5.0 × 10^5^, and 5.0 × 10^4^ CFU/mL using sterile phosphate buffer saline (PBS). Six groups of 20 healthy Chinese longsnout catfish (8.2~13.5 g) each were randomly assigned at a water temperature of 29 ± 1 °C. The challenge test was conducted using a 0.1 mL intraperitoneal injection for each fish. The control group was injected with an equal volume of sterile PBS. After artificial infection, clinical signs and mortality were monitored daily for 14 days, and dead fish were subjected to bacterial isolation and identification. The median lethal dose was calculated using the modified Karber’s method [31,32] as follows:

LD_50_ = lg^−1^[Xk − i(∑p − 0.5)]

“Xk” is the logarithmic value of the maximum dose;
“i” is the logarithmic dose difference between adjacent dose groups;
“∑p” is the sum of mortality rates for each dose group.

### 2.5. Antimicrobial Susceptibility Testing

Antimicrobial susceptibility testing (AST) of strain LL2208 was performed using the Kirby–Bauer disk-diffusion method. Antimicrobial drugs, including penicillin G (10 U), amoxicillin (20 µg), cephalexin (30 µg), ceftriaxone (30 µg), tetracycline (30 µg), doxycycline (30 µg), chloramphenicol (30 µg), florfenicol (30 µg), enrofloxacin (10 µg), ofloxacin (5 µg), norfloxacin (10 µg), gentamicin (10 µg), streptomycin (10 µg), tobramycin (10 µg), roxithromycin (15 µg), erythromycin (15 µg), sulfamethoxazole/trimethoprim (23.75/1.25 µg), sulfamethoxazole (300 µg), lincomycin (2 µg), vancomycin (30 µg), and fosfomycin (30 µg) were obtained from Hangzhou Microbial Reagent Co., Ltd. (Hangzhou, China). Strain LL2208 was inoculated into a Mueller–Hinton agar (MHA) plate and cultured at 28 °C overnight. The bacterial solution was diluted to 0.5 McFarland standard and streaked on MHA plates. The paper disks were placed on MHA plates, which were then incubated at 35 °C for 24 h to measure the diameter of the inhibition zone. The *Escherichia* coli strain ATCC 25922 was used as a quality control strain. AST was performed in triplicate. The susceptibility results were interpreted based on the Clinical Laboratory Standards Institute (CLSI) guidelines [33] and the manufacturer’s instructions.

### 2.6. Whole-Genome Sequencing and Bioinformatics Analysis

#### 2.6.1. Sequencing and Functional Genes Prediction

The genomic DNA of strain LL2208 was sequenced using PacBio technology (PacBio Sequel II sequencing platform) and assembled using Canu v1.5 and wtdbg v2.2 software to obtain the complete genome sequence. Prodigal 2.6.2 software (https://github.com/hyattpd/prodigal/wiki (accessed on 9 July 2023)) was used for gene prediction, and tRNAscan-SE and Barnap 0.7 software was used to predict tRNA and rRNA, respectively. IslandViewer 4 (http://www.pathogenomics.sfu.ca/islandviewer/ (accessed on 9 July 2023)) and PhiSpy (https://github.com/linsalrob/PhiSpy (accessed on 9 July 2023)) were used to predict the gene islands and pre-phages, respectively. Multilocus sequence typing (MLST), capsule type, and O antigen type of strain LL2208 were analyzed using the online tool PathogenWatch (https://pathogen.watch (accessed on 22 April 2024)).

#### 2.6.2. ANI and dDDH Analysis

The average nucleotide identity (ANI) is widely used to classify and identify bacteria by calculating the ANI values of prokaryotic genome sequences [34]. ANIu values were calculated between strain LL2208 and related species using the online tool OrthoANIu (https://www.ezbiocloud.net/tools/ani (accessed on 23 April 2024)). Digital DNA-DNA hybridization (dDDH) values between the LL2208 genome and the genomes of related species were analyzed using the Genome-to-Genome Distance Calculator 3.0 (https://ggdc.dsmz.de/ggdc.php (accessed on 24 April 2024)) [35].

#### 2.6.3. Virulence-Related Genes

The virulence-related genes in the genome of strain LL2208 were analyzed using VFanalyzer (http://www.mgc.ac.cn/cgi-bin/VFs/v5/main.cgi (accessed on 8 May 2024)). The virulence genes such as *impA*, *impA2*, *magA*, *peg*-344, *iroB*, and *iucB* were detected using PCR amplification and sequencing. For the PCR amplification, the Ex Taq™ Version 2.0 plus dye was used and the primers were listed in Table 1. The PCR conditions were as follows: pre-denaturation at 95 °C for 4 min, followed by 35 cycles (denaturation at 94 °C for 30 s, annealing at 56–59 °C for 30 s, and extension at 72 °C for 1 min), and final extension at 72 °C for 10 min. Amplified products were collected and sequenced by Sangon Biotech (Shanghai) Co., Ltd., Shanghai, China.

#### 2.6.4. Antimicrobial Resistance Genes

Antimicrobial resistance genes were predicted using the RGI 6.0.3 software (https://card.mcmaster.ca/analyze/rgi (accessed on 22 May 2024)) in the Comprehensive Antibiotic Resistance Database (CARD) with strict hit criteria.

#### 2.6.5. Collinearity Analysis

Collinearity analysis of different strains is important in revealing in-depth genetic variation. BRIG-0.95 software (https://sourceforge.net/projects/brig/ (accessed on 22 May 2024)) was used to obtain the collinearity relationship between strain LL2208 and closely related evolutionary strains [36], including strains WCHKV030666 [37] and 15WZ-82 [38].

## 3. Results

### 3.1. Bacterial Isolation and Identification

**Figure 1:**
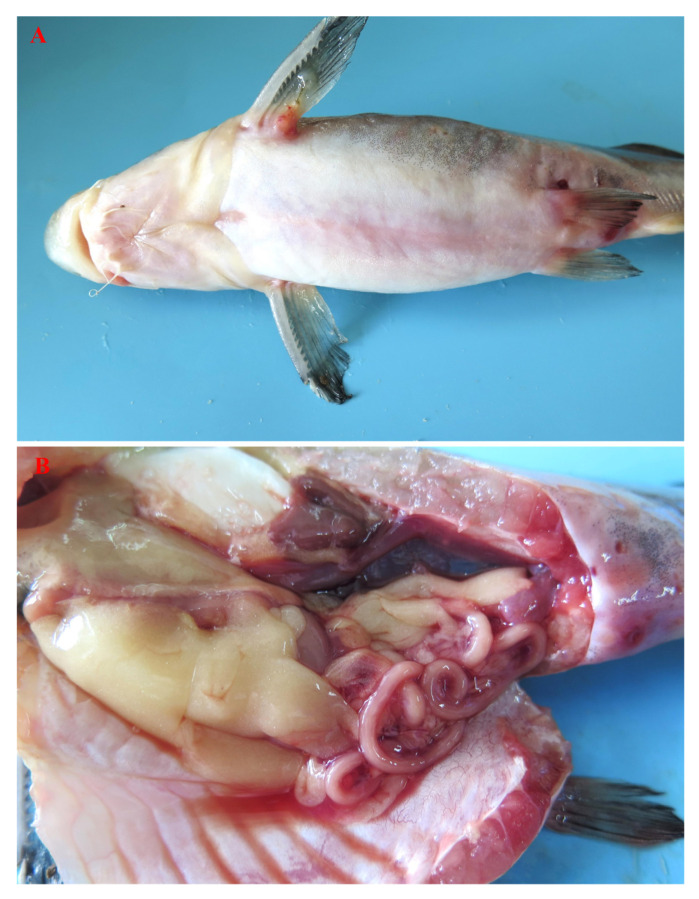
Clinical signs of naturally occurring disease in Chinese longsnout catfish. (**A**) Swollen abdomen, hemorrhage in the base of the fin, anal dilatation with hyperemia; (**B**) fluid accumulation in the abdominal cavity, and liver and kidney enlargement.

**Figure 2:**
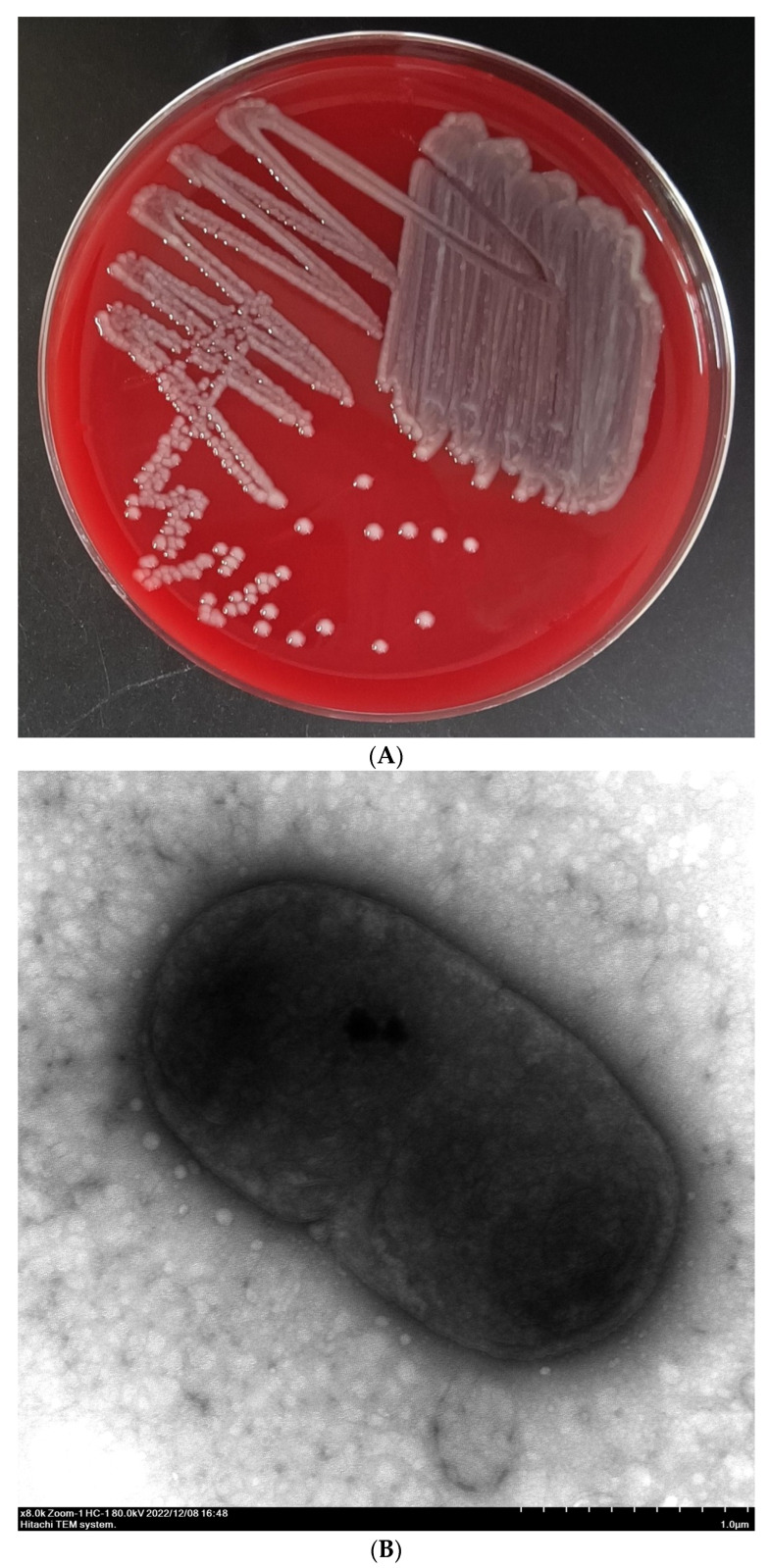
Morphological characteristics of the colony (**A**) and bacterium (**B**) of strain LL2208. Strain LL2208 was streaked on Columbia blood agar plate and incubated at 29 °C for 48 h. The bacterial morphology of strain LL2208 was observed using transmission electron microscopy (HT7800; Hitachi, Tokyo, Japan) with 6000× magnification.

Clinical signs of the naturally diseased Chinese longsnout catfish (100–600 g) included a swollen abdomen, hemorrhage in the base of the fin, anal dilatation with hyperemia, fluid accumulation in the abdominal cavity, and liver and kidney enlargement (Figure 1). Many bacteria with similar colony morphologies were isolated from the liver, spleen, and kidney tissues of diseased fish (N = 7). The colonies appeared moist and bumped, with regular edges, smooth surfaces, and a grey-white color (Figure 2A). The bacterial morphology of strain LL2208 was observed by transmission electron microscopy, indicating that the strain was thick, short, and rod-shaped, with a large number of extracellular products, filamentous pili, and outer membrane vesicles (Figure 2B).

**Figure 3:**
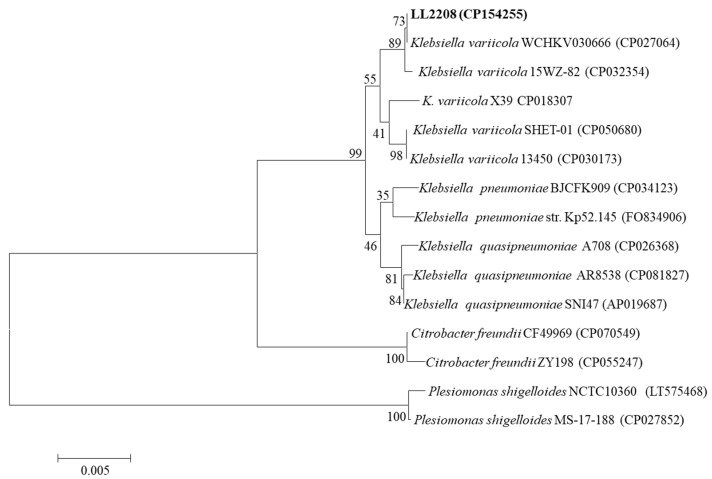
The phylogenetic tree constructed based on 16S rRNA gene sequences.

The 16S rRNA sequence of strain LL2208 was analyzed through Blastn online alignment, and the results showed that the sequence similarities between strain LL2208 and *K. pneumoniae* or *K. variicola* strains were ≥99.94%. A phylogenetic tree of the 16S rRNA sequences was constructed, suggesting that strain LL2208 and *K. variicola* strains were clustered into the same branch (Figure 3). Specific PCR detection showed that strain LL2208 belonged to *K. variicola*, with the PCR product sequence being 100% similar to that of the *K. variicola* strain 15WZ-82 (GenBank accession no. CP032354.1, located in 3,232,504 bp~3,232,992 bp), indicating that strain LL2208 could be classified as *K. variicola*.

### 3.2. String Test

**Figure 4:**
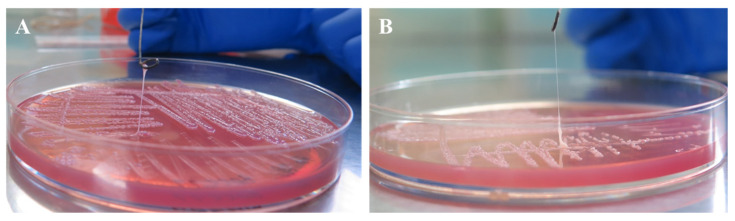
String test of strain LL2208 with initial strain (**A**) and two passages (**B**).

String tests of strain LL2208 showed that it produced a 2–4 cm viscous string after 18 h of cultivation on MacConkey agar plates (Figure 4). Moreover, strain LL2208 produced a >10 mm viscous string after two passages of cultivation, indicating that it was phenotypically hmvKv.

### 3.3. Biofilm Formation Ability

The biofilm assay showed that the OD_595_ of strain LL2208 was 1.41 ± 0.10, while the OD_595_ of the control group was 0.17 ± 0.01, indicating that strain LL2208 has strong biofilm formation ability (>4 OD_595c_), and it was an hmvKv strain.

### 3.4. Pathogenicity 

The major clinical signs of fish infected with strain LL2208 were slow swimming, fluid accumulation in the abdominal cavity, and liver and kidney enlargement. A large number of colonies with similar morphologies were isolated from the liver and kidney tissues of the infected fish, and the similarities of the 16S rRNA sequences between the isolated strains and strain LL2208 were 100%, indicating that the death of *L. longirostris* was caused by artificial infection. The fish in the control group showed no clinical signs or mortality during the observation period. The LD_50_ of strain LL2208 to *L. longirostris* was 7.92 × 10^7^ CFU/mL.

### 3.5. Antimicrobial Susceptibility

AST results showed that strain LL2208 was resistant to penicillins (penicillin, amoxicillin), macrolides (erythromycin and roxithromycin), amphenicols (chloramphenicol and florfenicol), lincosamides (lincomycin), and glycopeptides (vancomycin), while it was sensitive or intermediate to cephalosporins (cephalexin, ceftriaxone), tetracyclines (tetracycline and doxycycline), quinolones (enrofloxacin, norfloxacin, and ofloxacin), sulfonamides (sulfamethoxazole/trimethoprim and sulfamethoxazole), aminoglycosides (tobramycin, gentamicin, and streptomycin), and fosfomycin (Table 2), indicating that strain LL2208 is multidrug-resistant.

### 3.6. Genomic Sequencing and Bioinformatics Analysis

#### 3.6.1. Genomic Annotation and Features

The genome of strain LL2208 contained a circularized chromosome (5,557,050 bp) with no identifiable plasmids and predicted 5158 CDSs (with protein), 88 tRNAs, and 25 rRNAs (5S, 16S, 23S). The whole-genome sequence was deposited in GenBank under the accession no. CP154255. Six gene islands were identified using IslandViewer 4 (Table S1). However, no prophages were found in the LL2208 genome. According to Pathogenwatch analysis, the MLST, capsule type, and O antigen type were ST595, KL16, and O5, respectively.

#### 3.6.2. ANI and dDDH Analysis

The OrthoANIu values of strain LL2208 against *K. variicola* strains were 99.10% to 99.98%, with the highest OrthoANIu value (99.98%) compared with the WCHKV030666 strain (Table 3). Generally, an ANI value > 95% indicates the presence of the same species; thus, strain LL2208 belongs to *K. variicola*. The dDDH values of strain LL2208 compared to *K. variicola* strains were 92.80% to 100% (Table 3), and the DDH values were >70%, confirming the presence of the same species. Therefore, the LL2208 strain was identified as *K. variicola* and is highly similar to strain WCHKV030666.

#### 3.6.3. Virulence-Associated Genes

A large number of virulence-related genes were predicted in the LL2208 genome, such as adhesion-related genes *mrkABCDFHI* and *fimABCDEFGHIK*, iron absorption-related genes *iutA*, *entABCDEFS*, *fepABCDG*, and *iroE*/*iroN*, as well as the T6SS secretion system (Table 4). However, genes *rmpA* and *magA* were not predicted in the genome. The *rmpA*, *iroB*, and *peg*-344 were detected using PCR amplification and sequencing, whereas *impA2*, *magA*, and *iucB* genes were not (Figure S1).

#### 3.6.4. Antimicrobial Resistance Genes

A total of 30 antimicrobial resistance genes (including repeat genes) were predicted in the LL2208 genome based on CARD, including *β*-lactamase resistance genes, e.g., *bla*_LEN-16_, *pbp3*, *ompK37*, and *mdtQ*; glycopeptide resistance gene *vanG*; and fosfomycin resistance genes *fosA6* and *uhpT*. The LL2208 genome contained four categories of antimicrobial resistance genes according to the mechanism of antibiotic resistance: antibiotic efflux, antibiotic inactivation, changes in antibiotic target sites, and reduced antibiotic permeability (Table 5).

#### 3.6.5. Collinearity Relationship

**Figure 5:**
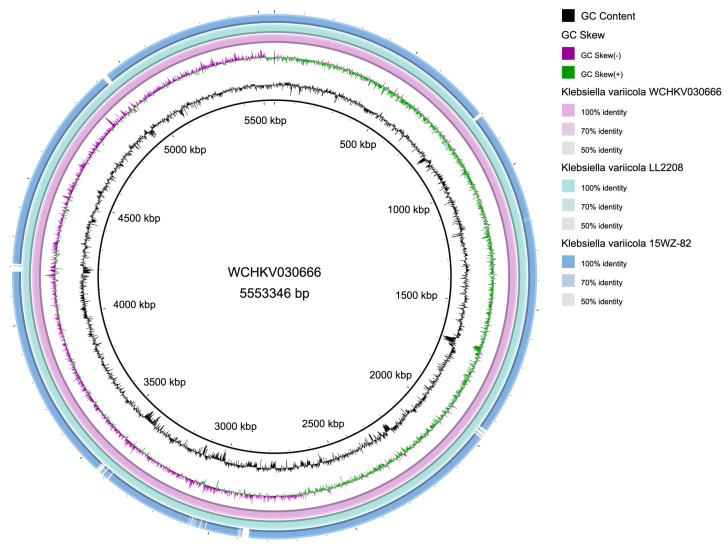
The relationship of genomic collinearity.

The results of collinearity analysis showed that strain LL2208 had a highly collinear relationship with strain WCHKV030666, and there was no gene rearrangement or inversion in the genome (Figure 5), indicating a high degree of similarity between these two strains. There was good collinearity between strain LL2208 and strain 15WZ-82; however, there were some gene deletions in the genome of strain 15WZ-82.

## 4. Discussion

*K. variicola* is a prevalent opportunistic pathogen that causes infections in humans and animals, including bloodstream infections, and urinary and respiratory-related diseases in humans [23]. It is divided into classical *K. variicola*, hmvKv, and hypervirulent *K. variicola* (hvKv). Some of these strains exhibit high infectivity and mortality rates [12]. Unlike *K. pneumoniae*, *K. variicola* strains have not been reported as a pathogen in aquatic animals. Moreover, limited information is available regarding the genetic background of *K. variicola* isolated from humans and fish.

In this study, strain LL2208 was isolated from the kidney of diseased Chinese longsnout catfish and was identified as *K. variicola* based on 16S rRNA, ANI, and DDH analysis, as well as specific PCR detection. Generally, the common methods for determining hypervirulent *K. pneumoniae* include the string test, capsular typing, iron uptake detection, and virulence phenotype detection. In this study, the string test showed that strain LL2208 was characterized as an hmvKv strain. The challenge test showed that strain LL2208 has moderate pathogenicity to the Chinese longsnout catfish, similar to that of *K. pneumoniae* KP0123 from *M. salmoides* with LD_50_ = 3.4 × 10^7^ CFU/mL [6]. Additionally, antimicrobial sensitivity tests showed that strain LL2208 is a multidrug-resistant strain resistant to antimicrobial agents, such as penicillins, macrolides, amphenicols, lincomycin, and glycopeptides. The results suggest that antimicrobial sensitivity tests should be performed before using antimicrobial agents to prevent and treat infection with *K. variicola* in fish to avoid the unreasonable use of antibiotics, leading to increases in drug-resistant strains.

The genome of strain LL2208 contains 30 resistance-related genes, such as the *β*-lactamase resistance genes, including *bla*_LEN-16_, *pbp3*, *ompK37*, and *mdtQ*, and the strain was resistant to penicillin but was sensitive to cephalosporins. It harbored *CRP*, *kpnE*, *kpnF*, *kpnG*, *kpnH*, and *H*-*NS*, contributing to macrolide resistance. Strain LL2208 harbored resistance gene *vanG*, which is responsible for vancomycin resistance, and it was resistant to amphenicols, which may be related to its carrying *rsmA*, *marA*, and *marR*. Furthermore, it carried fluoroquinolones/tetracycline resistance-related genes (*adeF*, *oqxA*, *marA*, and *marR*), disinfectant resistance-related genes (*leuO* and *qacG*), and fosfomycin resistance genes (*fosA6* and *uhpT*). However, strain LL2208 was not resistant to fluoroquinolones, tetracycline, and fosfomycin; perhaps these genes in the genome were associated with low-level resistance. Consistent with previous reports, *K. variicola* strains from humans or animals also carried many antimicrobial resistance genes; for example, *K. variicola* strain AHKv S01 isolated from dead chicken embryos carried antibiotic resistance genes, including *bla*_LEN-2_, *ampH*, *adeF*, *ompK37*, *kpnH*, *kpnG*, *kpnE*, *emrR*, *oqxA*, *baeR*, *mdtC*, *mdtB*, *marA*, *acrB*, *acrR*, *CRP*, *fosA2*, and *uhpT*, and this strain was resistant to penicillins, macrolides, and lincosamides [1], indicating that strains AHKv S01 and LL2208 have a similar phenotype and genotype of antimicrobial resistance. *K. variicola* strain KV093 was resistant to tircarcillin, piperacillin, oxacillin, norfloxacin, methicillin, linezolid, clindamycin, azithromycin, and amikacin, and it harbored *bla*_LEN-9_, *fosA6*, *ampH*, *pbp3*, *mcr*-9.1, *mcr*-3.5, *mdtABC*, *emrAB*, *oqxAB*, *mdtIJ*, and *qacA* [23]. Taken together with the current study results, this suggests that *bla*_LEN_, *pbp3*, *oqxA*, and *fosA* genes are common resistance genes of *K. variicola*.

The typical virulence factors of the *Klebsiella* genus include capsular polysaccharides, pili/fimbriae, iron-binding siderophores, lipopolysaccharides, outer membrane proteins, and T6SS [39]. Type I fimbriae are encoded by *fim* gene clusters and can bind to glycoproteins in epithelial cells, contributing to bacterial colonization; type III fimbriae are encoded by *mrkABCDF*, which is related to biofilm formation and invasion. Strain LL2208 contained type I and III fimbrial gene clusters, including *fimABCDEFGHIK* and *mrkABCDFHI*, implying that this strain has strong adhesion ability and biofilm formation ability [40]. Siderophores are closely related to hypervirulent *K. pneumoniae*, including aerobactin, enterobactin, yersiniabactin, and salmochelin [39]. In *K. pneumoniae*, enterobactin is the main siderophore for iron uptake, and salmochelin is common in hypervirulent *K. pneumoniae* (hvKp) strains; more than 90% of these are associated with pyogenic liver abscesses and effective infection in hvKp requires aerobactin [39]. Generally, siderophores are essential for the proliferation and pathogenicity of bacteria and can affect tissue localization and systemic dispersion. Strain LL2208 contained genes associated with the synthesis of salmochelin (*iroE* and *iroN*), enterobactin (*entABCDEFS* and *fepABCDG*), and aerobactin (*iutA*), indicating that it could synthesize salmochelin, enterobactin, and aerobactin, which may enhance its pathogenicity.

The virulence genes *rmpA*, *rmpA2*, *peg344*, *iroB*, and *iucA* can distinguish between hvKP and classical *K. pneumoniae* (cKP). Gene *rmpA* was not identified in the genome of strain LL2208. However, *rmpA*, *peg344*, and *iroB* were detected using PCR amplification and sequencing, suggesting that the hypermucoviscous phenotype of strain LL2208 was related to *rmpA*. Moreover, strain LL2208 may have low-copy plasmids, and the plasmid sequences were not assembled because the sequencing depth was insufficient. Strain LL2208 contained *rmpA*, *peg344*, *iroB*, and *iucA* genes (Table 4), suggesting that this strain was an hvKv strain based on the presence of virulence-associated genes.

*K. variicoa* strains are becoming a threat to public health worldwide. Recently, the number of reports on human hvKv strains has increased. A multidrug-resistant *K. variicoa* strain (ST17) resulted in a higher mortality rate (54.5%) in newborns [41]. The hvKv strain 21072329 isolated from an elderly patient belonged to the ST11-KL47 type and is resistant to carbapenems and third- and fourth-generation cephalosporins [42]. *K. variicola* strain wxkv2055, with ST595-KL16-O5 genotype, was isolated from human blood [43], consistent with the report that strain LL2208 also belongs to the same genotype. Furthermore, the genotype of *K. variicola* strain WCHKV030666 isolated from humans was ST595-O5, and the capsule type was similar to KL16 (wzi251) [37]. Moreover, the strains LL2208 and WCHKV030666 showed a highly collinear relationship, indicating they have a common ancestral lineage. A carbapenem-resistant *K. variicola* strain 15WZ-82 isolated from human sputum exhibited high-level virulence in mice and showed good collinearity with strain LL2208 [38]. These results indicate that strain LL2208 can potentially become an hvKv. *K. variicola*, a co-pathogenic bacterium found between humans and aquatic animals, can cause infection in farmed fish if the aquaculture water is contaminated with this strain. Moreover, *K. variicola*, as a potential foodborne pathogen, poses a risk to human health [44].

In this study, enrofloxacin and sulfonamides are recommended to prevent and control the infection of *K. variicola* in Chinese longsnout catfish according to the results of the antimicrobial sensitivity test. However, it is necessary to accelerate the research and development of prevention and control products for *K. variicola*, which belongs to the *K. pneumoniae* complex, to curb the spread of multidrug-resistant strains. For example, lytic phage was isolated from water as a biocontrol agent against multidrug-resistant *K. variicola*, inhibiting its growth in vitro and in vivo [45]. Oral treatment with live commensal *Bifidobacterium longum* 5^1A^ prevents detrimental lung inflammation caused by *K. pneumoniae* strains [46]. A previous report also showed that plant extracts could effectively inhibit the growth of *K. pneumoniae*, and peppercorn extracts were the most potent growth inhibitors [47]. Vaccines are a promising strategy for preventing infections caused by *K. pneumoniae* strains and reducing the spread of multidrug-resistant strains [48].

## 5. Conclusions

A multidrug-resistant hmvKv strain LL2208 with strong biofilm formation ability was isolated from diseased fish. Genome analysis revealed that the strain harbored antimicrobial resistance genes, including *bla*_LEN-16_, *ompK37*, *pbp3*, and *vanG*, and it contained numerous virulence-associated genes, including type I and III fimbrial gene clusters, siderophores, and T6SS. The genotype was ST595-KL16-O5. Comparative genomics analysis revealed strain LL2208 is highly similar to human-derived *K. variicola* strains, suggesting that the spread of *K. variicola* strains in diseased fish poses a potential risk to public health. Monitoring the epidemiology, pathogenicity, and antimicrobial resistance of *K. variicola* as an emerging pathogen in fish is crucial for disease prevention and safeguarding public health.

## Figures and Tables

**Table 1:** Primer sequences of virulence-associated genes.

Gene	Primer	Sequence (5′-3′)	Length (bp)
*rmpA*	KvrmpA-F	AACTGGACTACCTCTGTTTC	537
	KvrmpA-R	CTTGGCATGAGCCATCTTTCA	
*rmpA2*	rmpA2-F	CTTTATGTGCAATAAGGATGTT	447
	rmpA2-R	CCTCCTGGAGAGTAAGCATT	
*magA*	magA-F	TTAATGTCTTAGGGCCTTTGC	720
	magA-R	ATTGACCATATTGCTCCGTTG	
*peg-344*	peg344-F	GCTCTTGAAACTATCCCTCCA	388
	peg344-R	GGGCAATAACTCCCGTCCACT	
*iroB*	iroB-F	CCCGATCTCATCATCTACCCTCC	752
	iroB-R	GCCATTTCCGCCGCTACCTCT	
*iucB*	iucB-F	ACTTTCAGCGGTGGTTCTCCC	509
	iucB-R	GGTCAAAGGGTTGCCATGAATAG	

**Table 2:** Results of drug sensitivity tests of strain LL2208.

Class	Antimicrobial Agents	Breakpoints (mm) *			Zone Diameter (mm)	Sensitivity
		R	I	S		
Penicillins	Penicillin	≤13	14~16	≥17	6.0	R
	Amoxicillin	≤13	14~17	≥18	6.0	R
Cephalosporins	Cephalexin	≤14	15~17	≥18	22.5	S
	Ceftriaxone	≤19	20~22	≥23	35.5	S
Tetracyclines	Tetracycline	≤11	12~14	≥15	17.5	S
	Doxycycline	≤10	11~13	≥14	15.0	S
Amphenicols	Chloramphenicol	≤12	13~17	≥18	6.0	R
	Florfenicol	≤12	13~17	≥18	10.2	R
Quinolones	Enrofloxacin	≤15	16~20	≥21	19.0	I
	Ofloxacin	≤12	13~15	≥16	18.3	S
	Norfloxacin	≤12	13~16	≥17	15.6	I
Aminoglycosides	Gentamicin	≤14	15~17	≥18	18.2	S
	Streptomycin	≤11	12~14	≥15	15.3	S
	Tobramycin	≤12	13~14	≥15	19.1	S
Macrolides	Roxithromycin	≤13	14~22	≥23	6.0	R
	Erythromycin	≤13	14~22	≥23	6.0	R
Sulfonamides	Sulfamethoxazole/Trimethoprim	≤10	11~15	≥16	23.3	S
	Sulfamethoxazole	≤12	13~16	≥17	17.4	S
Lincosamides	Lincomycin	≤9	10~15	≥16	6.0	R
Glycopeptides	Vancomycin	≤9	10~15	≥16	6.0	R
Others	Fosfomycin	≤12	13~15	≥16	19.7	S

Notes: R, resistant; I, intermediate; S, susceptible. * The breakpoints were determined according to the Performance Standards for Antimicrobial Susceptibility Testing [33].

**Table 3:** OrthoANIu and dDDH analysis between LL2208 and reference *Klebsiella* strains.

Klebsiella Strains	GenBank Accession No.	OrthoANIu Value (%)	dDDH (%)
*K. variicola* WCHKV030666	CP027064	99.98	100
*K. variicola* 15WZ-82	CP032354	99.74	98.70
*K. variicola* 13450	CP030173	99.10	92.80
*K. pneumoniae* str.Kp52.145	FO834906	94.55	58.60
*K. pneumoniae* BJCFK909	CP034123	94.55	58.30
*K. quasipneumoniae* A708	CP026368	93.34	52.50

**Table 4:** Virulence-associated genes in the genome of strain LL2208 were predicted by VFanalyzer.

Class	Virulence Factors	Genes
Adherence	Type III fimbriae	*mrkA*, *mrkB*, *mrkC*, *mrkD*, *mrkF*, *mrkH*, *mrkI*
	Type I fimbriae	*fimA*, *fimB*, *fimC*, *fimD*, *fimE*, *fimF*, *fimG*, *fimH*, *fimI*, *fimK*
Efflux pump	AcrAB	*acrA*, *acrB*
Iron uptake	Aerobactin	*iutA*
	Ent siderophore	*entA*, *entB*, *entC*, *entD*, *entE*, *entF*, *entS*, *fepA*, *fepB*, *fepC*, *fepD*, *fepG*, *fes*
	Salmochelin	*iroE*, *iroN*
Regulation	RcsAB	*rcsA*, *rcsB*
Secretion system	T6SS-I	*clpV/tssH*, *dotU/tssL*, *hcp/tssD*, *icmF/tssM*, *impA/tssA*, *ompA*, *sciN/tssJ*, *tssF*, *tssG*, *vasE/tssK*, *vgrG/tssI*, *vipA/tssB*, *vipB/tssC*
	T6SS-II	*clpV*
	T6SS-III	*impF*
Autotransporter	Contact-dependent inhibition CDI system	*cdiA*
Fimbrial adherence determinants	Stc	*stcB*, *stcC*
	Sti	*stiB*
Magnesium uptake	Mg^2+^ transport	*mgtB*

**Table 5:** Genes associated with the antibiotic resistance of strain LL2208.

Resistance Mechanism	Antimicrobial Resistance Gene Family	Genes
Antibiotic efflux	MFS antibiotic efflux pump	*kpnG*, *kpnH*, *emrR*, *H-NS*, *leuO*
	RND antibiotic efflux pump	*CRP*, *rsmA*, *adeF*, *oqxA*, *baeR*, *marR*, *marA*
	SMR antibiotic efflux pump	*kpnE*, *kpnF*, *qacG*
	ABC antibiotic efflux pump	*lptD*, *msbA*
Antibiotic inactivation	Beta-lactamase	*LEN-16*
	Fosfomycin thiol transferase	*fosA6*
Antibiotic target alteration	phosphoethanolamine transferase	*eptB*, *arnT*
	glycopeptide resistance gene	*vanG*
	Elfamycin-resistant	*EF-Tu*
	Fosfomycin-resistant	*uhpT*
	Penicillin-binding protein mutations	*pbp3*
Reduced permeability to antibiotic	Outer Membrane Porin	*mdtQ*
	Reduced permeability to beta-lactams	*ompK37*

## Data Availability

The data are included in the article/referenced in the article.

## Supplementary Materials

The following supporting information can be downloaded at: https://www.mdpi.com/article/10.3390/pathogens13080647/s1, Figure S1: Virulence-associated genes detected by PCR; Table S1: Gene islands in the genome of strain LL2208 predicted by IslandViewer 4.
