# Supplementary material for: Hypermucoviscous Multidrug-Resistant Klebsiella variicola Strain LL2208 Isolated from Chinese Longsnout Catfish (Leiocassis longirostris): Highly Similar to Human K. variicola Strains
Source: Pathogens. 2024 Jul 31;13(8):647. doi: 10.3390/pathogens13080647 (PMC11356897; doi:10.3390/pathogens13080647)
Supplement: Supplementary file 1 [file pathogens-13-00647-s001.zip › pathogens-3092748-supplementary.pdf]

Hypermucoviscous Multidrug-Resistant *Klebsiella variicola* Strain LL2208 Isolated from Chinese Longsnout Catfish (*Leiocassis longirostris*): Highly Similar to Human *K. variicola* Strains

Qingyong Li [1], Xin Yu [1], Lin Ye [1], Tongyu Hou [1], Yi Liu [1], Guiming Liu [1], Qing Wang [2,3] and Defeng Zhang [2,3,*]

[1] Fisheries Research and Extension Center of Huizhou, Huizhou 516055, China; byqyli@163.com (Q.L.); 27763967@126.com (X.Y.); 13528069240@139.com (L.Y.); houtongyu@hotmail.com (T.H.); 3825431221@139.com (Y.L.); 13928333187@189.com (G.L.)
[2] Key Laboratory of Fishery Drug Development, Ministry of Agriculture and Rural Affairs, Pearl River Fisheries Research Institute, Chinese Academy of Fishery Sciences, Guangzhou 510380, China; wangqing@prfri.ac.cn
[3] Guangdong Provincial Key Laboratory of Aquatic Animal Immunology and Sustainable Aquaculture, Pearl River Fisheries Research Institute, Chinese Academy of Fishery Sciences, Guangzhou 510380, China
* Correspondence: zhangdefeng@prfri.ac.cn

Table S1. Gene islands in the genome of strain LL2208 predicted by IslandViewer 4.

Gene Islands	Positions in Genome
GI_1	1,191,331 bp – 1,203,694 bp
GI_2	1,943,658 bp – 1,967,036 bp
GI_3	3,016,227 bp – 3,040,252 bp
GI_4	3,172,831 bp – 3,182,967 bp
GI_5	4,185,931 bp – 4,215,920 bp
GI_6	4,242,977 bp – 4,254,623 bp

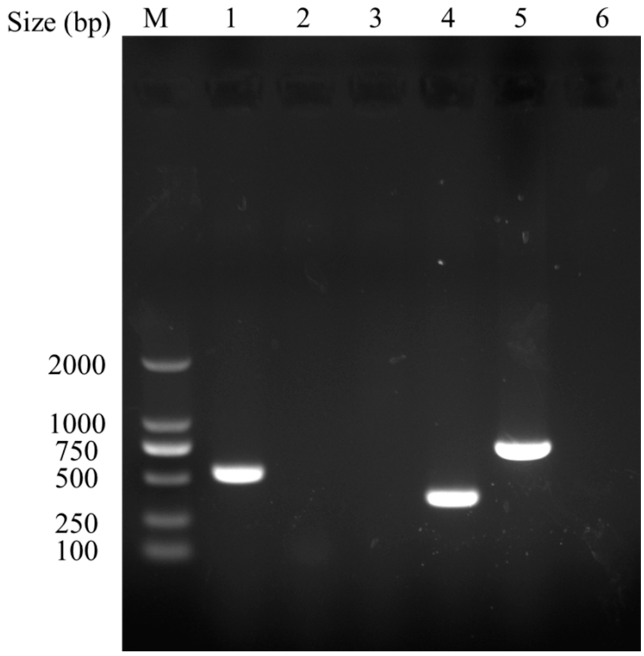

Figure S1. Virulence associated genes were detected by PCR.
Lane M, DNA markers (DL2000); Lane 1, *rmpA*; Lane 2, *rmpA2*; Lane 3, *magA*; Lane 4, *peg-344*; Lane 5, *iroB*; Lane 6, *iucB*.
